# The Associated Decision and Management Factors on Cattle Tick Level of Infestation in Two Tropical Areas of Ecuador

**DOI:** 10.3390/pathogens11040403

**Published:** 2022-03-26

**Authors:** Valeria Paucar, Ximena Pérez-Otáñez, Richar Rodríguez-Hidalgo, Cecilia Perez, Darío Cepeda-Bastidas, Jorge Grijalva, Sandra Enríquez, Susana Arciniegas-Ortega, Sophie O. Vanwambeke, Lenin Ron-Garrido, Claude Saegerman

**Affiliations:** 1 Instituto de Investigación en Zoonosis (CIZ), Universidad Central del Ecuador, Quito 170521, Ecuador; avpaucar@uce.edu.ec (V.P.); xfperez@uce.edu.ec (X.P.-O.); rrodriguez@uce.edu.ec (R.R.-H.); ienriquez@uce.edu.ec (S.E.); ljron@uce.edu.ec (L.R.-G.); 2 Research Unit of Epidemiology and Risk Analysis Applied to Veterinary Sciences (UREAR-ULiège), Fundamental and Applied Research for Animals & Health (FARAH) Center, Faculty of Veterinary Medicine, University of Liege, 4000 Liège, Belgium; sophie.vanwambeke@uclouvain.be; 3 Georges Lemaitre Centre for Earth and Climate Research, UCLouvain, 1348 Louvain-la-Neuve, Belgium; 4 Facultad de Medicina Veterinaria y Zootecnia, Universidad Central del Ecuador, Quito 170521, Ecuador; bcperez@uce.edu.ec (C.P.); jgrijalva@uce.edu.ec (J.G.); 5 Facultad de Ciencias Agrícolas, Universidad Central del Ecuador, Quito 170521, Ecuador; dacepedab@uce.edu.ec; 6 Facultad de Geología, Minas y Petróleo, Universidad Central del Ecuador, Quito 170521, Ecuador; srarciniegas@uce.edu.ec

**Keywords:** acaricide, cattle, Ecuador, protective factor, risk factor, tick, tick-borne diseases, tropical

## Abstract

Decision-making on tick control practices is linked to the level of knowledge about livestock farming and to the social context in which individuals practice them. Tick infestation is one of the main problems in tropical livestock production. The objective of this study was to characterize tick-control related practices in two tropical livestock areas and their potential association with the level of tick infestation. A total of 139 farms were included in this survey. To determine this association, a multivariate logistic regression model was used. A stepwise model selection procedure was used and model validation was tested. Cattle husbandry as a main activity, the use of external paddocks, the use of amitraz, and the lack of mechanization on the farm were related with high tick infestation. On the other hand, owner involvement in the preparation of acaricide solution was identified as a protective factor against high tick infestation. At animal level, age (old), body condition status (thin), and lactation were also associated with high tick infestations, while *Bos primigenius indicus* cattle and their crosses reduced the probability of high tick infestations. The factors studied, such as herd size, education level of the owners, and veterinary guidance, varied from farm to farm. Nonetheless, these differences did not generate changes in the level of tick infestation. According to the area under the receiver operating characteristic curve (AUC-ROC), the model at farm level predicts a high level of infestation, with an accuracy of 72.00% and high sensitivity. In addition, at animal level, crossbreeding with indicus cattle and breeding selection for host resistance will be useful against high tick infestation. Likewise, the implementation of programs of capacitation and research on tick control for farmers, cowboys, and vets in these areas is necessary.

## 1. Introduction

Livestock is a major economic activity in Ecuador. It contributes substantially to local nutrition, providing milk, meat, and derivatives that are in high demand by the population [1]. The agriculture sector, including livestock farming, represents around 7.80% of the total of Ecuador’s gross domestic product [2]. Ecuadorian cattle population is about 4.3 million heads, from 280,000 cattle farms nationwide [3]. Due to Ecuador’s location, most of the country, except for certain parts of highlands (places above 2500 m), experiences a humid tropical climate [4,5], providing favorable environmental conditions for the development of ectoparasites such as ticks. In fact, more than 75.00% of cattle herds are found in areas either infested or potentially infested with ticks [6].

Tick infestation causes significant economic losses in the livestock industry. Ticks transmit a wide range of pathogens that can cause tick-borne diseases (TBDs). The most important TBDs of cattle in Ecuador are anaplasmosis caused by rickettsia of the genus *Anaplasma* [7] and babesiosis caused by protozoa of the genus *Babesia* [8]. In addition to spreading pathogenic microorganisms, ticks cause weight loss, reduced milk production, and cause skin injures that can lead to secondary bacterial and fungal infections and even myasis (*Cochlomyia hominivorax*) [9,10,11]. Additional losses include the cost of treatment for clinical cases and the expenses derived from the indiscriminate use of acaricides for tick control. Likewise, the indiscriminate use of chemical compounds has increased the problem of tick multiresistance to acaricides, which has already been reported in Ecuador [11]. 

The livestock production present in tropical areas is extensive, with a low level of mechanization used, and grazing is the main source of food for animals [12]. Consequently, to increase milk production, farmers tend to introduce exotic breeds (*Bos primigenious taurus*), which often are susceptible to TBDs. In addition, for improving farm profitability, natural ecosystems are incorporated into production, clearing forests to plant non-native pasture, in order to expand the agricultural frontier [13,14]. These environmental and host modifications have had a major impact on the ecology of these parasites, causing the encounter rate between tick and host to be higher, leading to an increase in tick infestation [15,16]. However, decisions on tick control practices are usually linked to the level of knowledge about livestock farming and to the social context in which individuals practice these strategies [17]. Understanding the reasons that lead farmers to use particular control measures will contribute to holding back the advance of the threat that acaricides pose to the environment and public health and also increase farm productivity. 

The three objectives of this study were to (i) describe two tropical dairy production areas located on the eastern and western foothills of the Ecuadorian Andes; (ii) relate tick control practices at animal and farm levels on the level of tick infestation; and (iii) identify tick species infesting cattle in these areas.

## 2. Results

### 2.1. Tick Species

In total, 1905 adult ticks were collected from 133 farms, 1345 ticks (70.60%) were females and 560 (29.40%) were males. In six farms, we did not find ticks on the animals examined. Tick prevalence in farms was estimated to be 95.70% (95% confidence internal, CI: 90–98). Table 1 shows the number of farms with tick presence and the tick species reported. Four species of ticks were morphologically identified. *Rhipicephalus microplus* (female: 1328; male: 553) being the most common species in the Northwest of Pichincha and Quijos river valley.

### 2.2. Characteristics of Farming and Tick Control

A total of 139 farms were visited, 72 in the Quijos river valley, and 67 in the Northwest of Pichincha province (Table 2). According to the number of cattle, most of the farms visited in the two areas were medium farms (21 to 70 cattle; 54.17% in Quijos river valley, and 64.18% in Northwest of Pichincha), followed by small farms (1 to 20 cattle) in the Quijos river valley (38.89%) and large farms (more than 70 cattle; 23.88%) in Northwest of Pichincha. The principal activity in those areas is cattle husbandry, with a focus on milk production. Agriculture occupies the second place, being practiced at 22.20% in Quijos river valley and 37.31% in Northwest of Pichincha. In 19.44% (Quijos river valley) and 32.84% (Northwest of Pichincha) of farms these two activities are practiced jointly. 

With respect to the education level, the percentage of farmers with university education was higher in Northwest of Pichincha (29.85%) than in Quijos river valley (9.72%). In the two areas, most of the farms are non-mechanized (65.28% and 56.72%, respectively). However, the number of mechanized farms is twice as high (35.82%) in Northwest of Pichincha compared to Quijos river valley (16.67%). Only 43.06% in Quijos river valley and 37.31% in Northwest of Pichincha have a storage space to keep veterinary drugs. All the farms use grazing as the feeding method. In addition, 31.94% in Quijos river valley and 43.28% in Northwest of Pichincha cut and carry pasture, and this is mainly used to feed the lactating dairy cows. Fifty percent (36 out of 72 farms) of the farms in Quijos river valley and 25.37% (17 out of 67 farms) of the farms in Northwest of Pichincha do not have sufficient feeding paddocks in the total area on the farm and have to use external farm paddocks to feed their cattle. Most of the external paddocks in Quijos river valley are owned (63.89%) by the farmer but, in Northwest of Pichincha most of these are rented (64.71%), and the farmers pay an annual fee for their use.

This survey revealed that most cattle farms in Quijos river valley (87.50%) have veterinary support. Of these farms, 81.43% are managed by public veterinarians. In Northwest of Pichincha, in the majority of farms (55.22%) do not have the accompaniment of a veterinarian. On farms with veterinary accompaniment (44.78%), this service is private in half of the cases (50.91%).

All farms (100.00%) in the Quijos river valley use chemical treatment for tick control, and in the Northwest of Pichincha 95.52% used it. Only a few farms (4.48%) did not use chemical control, because they prefer uncommon control methods such as bath spray with entomopathogenic fungus, medicinal plants (*Azadirachta indica*), sulfocalcic broth, or sulfur supplementation in the diet. In most cases, the frequency of application of an acaricide treatment is less than once per month. The main method of application of acaricides was spraying with a hand sprayer (96.32%). In spray solution, the most commonly used acaricides were amides and organophosphates. Amides (amitraz) were used in 80.56% and 67.16% of the farms in Quijos river valley and Northwest of Pichincha, respectively. Organophosphates were used by 50.75% of all farms in Quijos river valley and 72.22% of the farms in Northwest of Pichincha. Ivermectin (macrocyclic lactone) was also commonly used for tick control by 77.78% of farms in the Quijos river valley and 86.57% in the Northwest of Pichincha (Table 2). This principle was administered parenterally (subcutaneously); however, in 19.65% and 6.90% of the farms in the Quijos river valley and Northwest of Pichincha it was applied topically (spraying). Among the farmers using ivermectin, 53.76% (Quijos river valley) and 50.00% (Northwest of Pichincha) use it on milking cattle. Although the two zones have different herd sizes and different levels of mechanization, both zones had similar percentages of high tick infestation (Quijos river valley with 41.67%; Northwestern of Pichincha with 40.29%) (*p*-Value > 0.05).

### 2.3. Tick-Infestation Associated Factors at Farm Level

Forty-one percent (95% CI: 32.84–49.68) (57/139) of farms had a high level of tick infestation. The variables included in the model are shown in Table 3. The initial model included 26 variables with (AIC: 206.40). The final model included eight factors significantly associated with high infestation level of ticks at farm level, selected for having the smallest value of AIC (AIC: 181.15). 

The final logistic regression model is presented in Table 4. The results of the model showed that cattle husbandry as the principal economic activity has a positive association with high levels of tick infestation. The OR when raising animals is the principal activity was 3.96 (95% CI: 0.97–16.10; *p*-Value 0.053, marginally significant) times higher than when cattle husbandry is not the principal activity. Absence of mechanization on the farm has a positive association (risk explanatory variable) with high tick infestation. Indeed, semi-mechanized (OR = 4.48 with 95% CI: 1.02–19.53) and non-mechanized farms (OR = 5.11 with 95% CI: 1.14–22.86) had higher odds ratio to have high tick infestation than the mechanized farming.

When the acaricide spray was prepared by the owner, there was less chance (OR = 0.19 with 95% CI: 0.06–0.061) of having animals with high tick infestation in the farms, in comparison with situations where the solution was prepared by employees.

### 2.4. Overall Weighted Score at Farm Level and Area under the Receiver Operating Characteristic Curve

The factors associated with a *p*-Value ≤ 0.10 in the final model (see Table 4) were used to calculate the weighted score. This threshold was used because of the relatively low number of farms sampled. Six covariates were aggregated as a unique overall weighted score (OWS) by farm, using the following formula:

  OWS = [(Presence_a = 1) × (OR_a)] + [(Presence_b2 = 1) × (OR_b2)] +  [(Presence_b3 = 1) × (OR_b3)] + [(Presence_c = 1) × (OR_c)] + [(Presence_d = 1) × (OR_d)] + [(Absence_e = 1) × (1/OR_e)] + [(Presence_f = 1) × (OR_f)](1)

where a = cattle husbandry is the principal activity; b2 = semi-mechanized farm; b3 = mechanized farm; c = farm with external paddocks; d = farm with veterinary support; e = the person who prepares the acaricide solution is the owner; f = use of amitraz.

With this formula, the minimum and the maximum theoretical values of the OWS are 5.11 and 21.08. The probability that a farm has a low or high level of tick infestation as a function of the result of the OWS is represented in Table 5.

**Figure 1:**
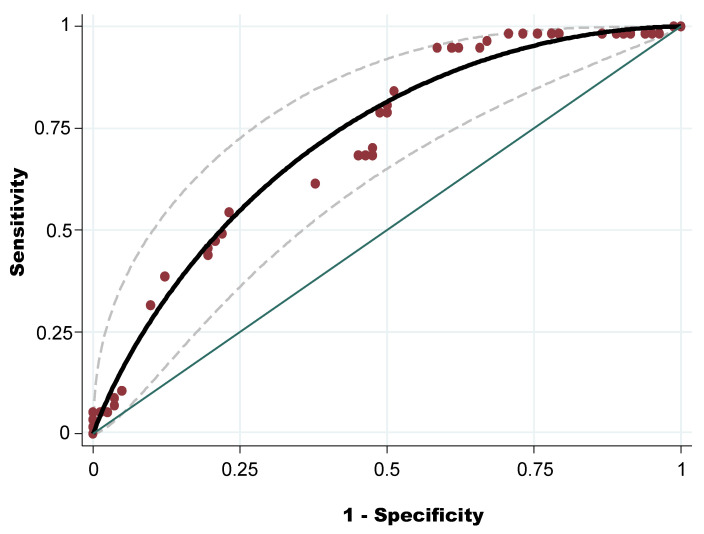
Receiver operating characteristic curve of the overall weighted score of a high level of tick infestation at farm level.

The diagnostic discriminatory power of OWS was assessed by calculating the AUC-ROC (Figure 1). The AUC-ROC was 0.72 (95% CI: 0.63–0.80), with standard error = 0.043. Using the Youden index (0.34), the best cut-off to discriminate the level of tick infestation (high and low level) was OWS = 11.65. Applying this cut-off, the sensitivity was 94.74 and the specificity was 41.46%.

### 2.5. Tick-Infestation Associated Factors at Animal Level

The covariates included in the analysis for tick infestation at animal level are shown in Table 6. In the univariate analysis six covariates were included. The sex of animals was discarded, given that only 27 out of 826 animals were males. For the final model, four covariates were associated with a high level of infestation at the animal level. 

The final logistic regression model is presented in Table 7, and the results of the multivariable binary logistic regression model showed that cattle breed: Crossbreed: *B. primigenious taurus* × *B. primigenious indicus* (OR = 0.547 with 95% CI: 0.546–0.548) and *B. p. indicus* (OR = 0.539 with 95% CI: 0.538–0.540) were protective factors against a high level of infestation. In comparison to young animals, young adult and adults over 7 years old had an OR = 1.050 (95% CI: 1.048-1.051) and OR = 1.480 (95% CI: 1.478–1.482), respectively. Cows in lactating status had an OR = 2.287 (95% CI: 2.283–2.900) in comparison to other categories. Finally, for body condition status, good (OR = 1.21 with 95% CI: 1.21–1.21) and thin (OR = 1.992 with 95% CI: 1.990–1.995) conditions were risk explanatory variables for high level of infestation in comparison to fat animals.

### 2.6. Overall Weighted Score (OWS) at Animal Level and Area under the Receiver Operating Characteristic Curve

The risk and protective explanatory variables with a *p*-Value ≤ 0.05 (see Table 6) were used to calculate the weighted score. Finally, four covariates were aggregated as a unique overall weighted score (OWS) by animal, using the following formula:

OWS = [(Absence_g1 = 1) × (1/OR_g2)] + [(Absence_g1 = 1) × (1/OR_g3)] +[(Presence_h = 1) × (OR_h2)] + [(Presence_h3 = 1) × (OR_h3)] + [(Presence_i = 1) × (OR_i)] +[(Presence_j2 = 1) × (OR_j2)] + [(Presence_j3 = 1) × (OR_j3)](2)

where g1= *B. primigenious taurus* g2= breed is a Crossbreed: *B. primigenious taurus* × *B. primigenious indicus*; g3 = breed is *B. primigenious indicus*; h2 = young adult animal; h3 = adult over 7 years old; i = lactating dairy cows; j2 = animal with good body condition status; and j3 = animal with thin body condition status. 

With this formula, the minimum and the maximum theoretical values of the OWS were 0.00 and 7.59.

**Figure 2:**
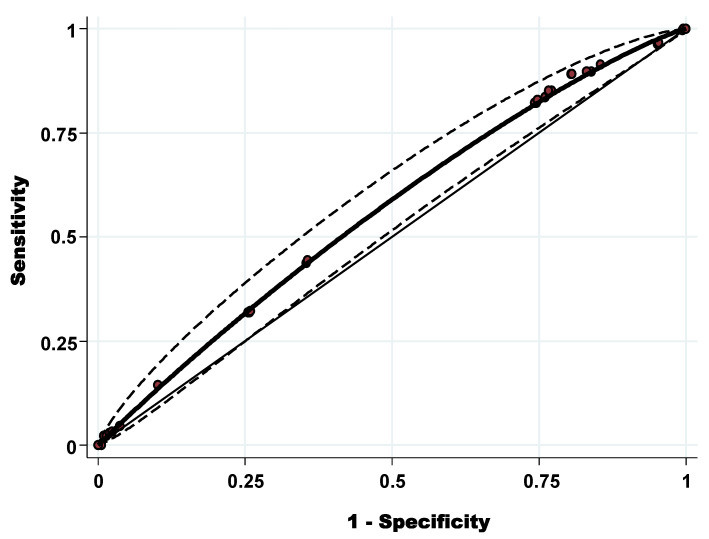
Receiver operating characteristic curve of the overall weighted score of high level of tick infestation at animal level.

The diagnostic discriminatory power was assessed by calculating the AUC-ROC (Figure 2). The AUC-ROC was 0.56 (95% CI: 0.52–0.60) with standard error = 0.021. Using both the Youden index (0.087) the best cut-off to discriminate the level of tick infestation (high and low level) was OWS = 3.34. Applying this cut-off, the sensitivity was 89.14%, and the specificity was 19.54%.

## 3. Discussion

*R. microplus* was the most collected and identified tick in both zones in this study, which confirms that it is the most common species on cattle. *I. boliviensis, I. montoyanus,* and *A. mixtum* were also identified on a few farms. Previous studies carried out in Ecuador have determined the presence of the *R. microplus* tick in Santo Domingo de los Colorados, Los Bancos, and Napo province [18,19,20,21,22,23,24]. Unpublished results obtained from the ‘Encuesta Nacional de Brucelosis, Tuberculosis y Garrapatas’, reported *R. microplus* as the most abundant species in tropical and subtropical areas of Ecuador. Nava et al. [25] and Aguilar-Domínguez et al. [26] reported the presence of *A. mixtum* larvae in the vegetation of coastal Ecuadorian localities. In addition, Guillén and Muñoz [20] identified *Amblyomma* spp. and *Ixodes* spp. on cattle at Santo Domingo de los Colorados. On the other hand, studies carried out in different parts of the Amazonia region have reported the presence of *I. boliviensis* [27] and *I. montoyanus* [28] on cattle.

In Ecuador, according to reports of several projects carried out by the Institute of Research in Zoonoses (unpublished data), *Amblyomma* *maculatum*, *Amblyomma ovale*, *Haempahysalis juxtakochi*, and *Dermacentor nitens* were also present on cattle. Species of the genus *Amblyomma* were reported by Enriquez et al. [29], Voltzit [30], and Maya et al. [22], who reported the presence of *Amblyomma coelebs*, *Amblyomma triste,* and *Amblyomma cajennense,* respectively, on cattle. 

When looking at the results of the number of farmers with university studies, mechanized farms and herd size (farms with more than 70 animals), it is evident that cattle husbandry is a more developed activity in the Northwest of Pichincha compared to the Quijos river valley. All this can be associated to the fact that the two zones had a different historical trajectory. The Northwest of Pichincha began to practice cattle husbandry over 50 years ago. This activity has expanded over time in relation to its location in a transit zone between the Coast region and the Highlands region, which facilitates the entrance of animals from other areas and the exit of livestock products to their destinations; becoming an area that supplies livestock products to the capital of Ecuador located in the Highlands region, and providing more than 200,000 L of raw milk per day [31,32,33]. On the other hand, cattle husbandry in the Quijos river valley produces 55,000 L of milk per day [33,34] and is a family activity that grew thanks to the construction of roads to the Amazon region for oil exploitation between 1968 and 1972 [35]. In addition, with the incursion of the multinational company Nestlé, cattle husbandry displaced agricultural production, which was previously the main source of income in the area [34,36,37]. Although the two zones had different herd sizes, farm mechanization, and education levels, these two zones had similar percentages of high tick infestation (Quijos river valley with 41.67%; Northwestern of Pichincha with 40.29%). This finding shows that some factors may not have been considered. Although the climate is very similar in both areas, and the sampling was done in the rainy season, the dry season in Northwestern of Pichincha is longer.

Regarding tick control methods, both zones mainly use chemical control. The range of acaricide products available on the market is wide. However, the site of action does not have much variety, as it can be seen that most of the acaricides used in this study belong to one of these families amide, organophosphate, pyrethroid, macrocyclic lactone, and phenylpyrazolone, whose mode of action is at the level of the nervous system [38,39]. The only acaricide with a different mode of action is fluazuron (benzoylphenyl urea). This acaricide is relatively new on the market. It is applied as a pour on that affects the molting process. However, it is expensive and has a long residual life in meat and milk [40,41]. The limited use of this acaricide in the study areas is associated with its price. Ivermectin (macrocyclic lactone) also generates residues in milk and meat for several weeks after application [41], but it is used by 77.78% of farmers in the Quijos river valley and 86.57% in the Northwest of Pichincha. It is also used in lactating cows. 

Acaricides were generally applied using hand sprayers, and the most usual acaricide applied in this survey was amitraz, an acaricide used extensively around the world, which entered in Ecuador in the 1960s [42]. Spraying consists of dissolving the correct dose of a wettable powder or flowable product in water [43]. However, this step is not followed by all farmers. Inadequate acaricide preparations (under-dosing or overdosing) and misapplications lead to the development of resistance [43,44], which has already been reported in Ecuador [11,22]. This fact would explain why farmers reduce the time between the treatments to less than one month in both zones (Quijos river valley, 80.56% and in Northwest of Pichincha, 67.16%). Bianchi et al., in 2003, [45] and Rodriguez-Vivas et al. in 2018 [39] reported that farmers used to apply control methods for ticks every month or whenever they observe a significant infestation level. Decreasing the interval between treatments is the first reaction when farmers observe that acaricide does not have the expected effect. Other common acaricides used are organophosphates, which despite being chemicals with a variable toxicity that can range from highly toxic to slightly toxic, and which have already been documented to cause neurological damage, are being used by farmers in the Quijos river valley in 77.78% and Northwest of Pichincha with 50.75% of farms. This is associated with the lack of knowledge that farmers have about the dangers of these acaricides. In addition to the fact that they are products freely sold in Ecuador, they do not need veterinary prescription [46], despite being banned in 32 countries (dichlorvos and trichlorfon) due to their harmful properties for the health and the environment [47].

At the animal level, the presence of crossbreed (*B. primigenious taurus* × *B. primigenious indicus*) and *B. primigenious indicus* breeds were protective to high levels of infestation in comparison with *B. primigenious taurus* cattle. This is because *B. primigenious indicus* cattle and their crossbreeds are genetically more resistant to ticks, which makes them adaptable to tropical climates [48,49,50]. The tolerance of *B. primigenious indicus* cattle is due to the coexistence and co-evolution of zebu cattle originating from Asia with *R. microplus* species also originating from the Asian continent, while European breeds (*B. p. taurus*) are more susceptible because they were less exposed in this evolution process, in addition to the fact that this type of cattle has thinner skin [14,51,52,53]. This is one of the main reasons why cattle breeds such as Jersey and Holstein have a higher level of tick infestation in tropical zones. 

Cattle in the studied areas with ‘good’ and ‘thin’ body conditions have 1.21 and 2.00 higher odds ratio of high level of tick infestation than cattle with ‘fat’ body condition. This is due to the fact that low nutrition causes metabolic, endocrine, and immunological consequences that increase parasitism [54]. These results are consistent with those reported by Sutherst et al. [55], Tolleson et al. [56], and Abbas et al. [38], who associated poor nutrition in cattle with increased tick burdens. In addition, a study in sheep found that lean sheep had 50.00% more ticks than fat sheep [55]. However, this is a factor to be taken with caution, as the worse body condition is also a consequence of high tick infestation. An average of 40 ticks per day per animal may cause losses around of 20 kg of weight per year [57,58].

Another factor associated with a high level of tick infestation was lactation, these animals had almost three times higher risk (OR = 2.29) of a high-level infestation compared to an animal that was not in production. This could be related to the fact that cows in this period have a high level of prolactin and progesterone, altering their immune system and making them more susceptible to infection. Indeed, infestation added to production stresses, such as pregnancy or lactation, decrease resistance to infection [59,60,61,62]. Additionally, dry cows can be treated with ivermectin at higher doses, given the lifting of restrictions for ivermectin usage during the milking period.

Animal age also constitutes a risk explanatory variable; young adults (OR = 1.05) and adults over 7 years (OR = 1.48) animals had a higher risk of having high tick infestation than young animals. This result is consistent with the work of Swai et al. in 2005 [63] and Rehman et al. in 2017 [61], who found that mature animals had a higher chance of carrying ticks compared to calves. The low infestation in young cattle is related to maternal immunity transmitted during suckling, maternal grooming, management, and also because in young cattle, farmers avoid free grazing, while the reduced size of calves reduces the area of contact with ticks [64,65].

Farm mechanization was an important preventive factor for high level of tick infestation. The odds ratio of high tick infestation on cattle from semi-mechanized (OR = 4.48) and non-mechanized (OR = 5.11) farms was higher, compared to mechanized farming. This result is consistent with studies in which farms furnished with cattle handling systems were at less risk of having a higher tick infestation than unequipped farms; this fact has also been observed in farms with poor facilities, where incorrect use of acaricides led to acaricide resistance [66,67]. 

The lack of paddocks for livestock feeding causes farmers to mobilize their animals to external paddocks. Although this practice helps to maintain the production of cattle, the use of external paddocks increased the risk for high tick infestation by 2.08 times, with regard to the farms that did not move animals outside the farm. Even though these paddocks can be owned or rented, in most cases they are rented for only few months of the year and not only to one farm, but to several farms during the year. Causing them to be used by different animals than can easily transport ticks between farms. Studies carried out by Heath in 2016 [68] and Zannou et al. in 2020 [69] found that herd movements around and between farms and pasture management can all also have a bearing on the presence of high level of tick infestation in the animals and in the occurrence or progress of tick-borne diseases (TBD).

The person who prepares the solution for the acaricide spray is a critical factor for the risk to present a high level of tick infestation. When the acaricide solution was prepared by the owner, there was a lower level of tick infestation (OR = 0.19) compared to farms where the spray solution was prepared by employees. This could be related to the differences in the level of education between owners and employees. Additionally, when there are training events, the person who attends is in most cases the owner. The insufficient knowledge and lack of training leads to under or over-dosing acaricides, which results in decreased acaricide efficacy and an increase in resistant tick populations [42,66,70]. 

Cattle husbandry is an important activity in the studied areas. However, the practice of cattle raising as the main activity was a significant explanatory variable for the presence of high level of tick infestation (OR = 3.96). This fact could be associated with several causes; among them, cattle raising is an activity that has been inherited from generation to generation [71]; therefore, correct and incorrect knowledge about the use of acaricides have also been transmitted between generation, fostering resistance to the commonly used acaricides [11,21,42]. Dairy farmers in Ecuador do not have access to research or tools to implement an integrated tick management control, which is based on the appropriate combination of at least two control tools as animal management practices, selection of cattle breeds resistant to ticks, use of plant extracts, pasture management, vaccination, or biological control [39]. The implementation of these tools requires economic resources, which are in part limited because the lack of clear government policies for the establishment of basic milk prices [72]. Likewise, innovative control strategies are needed, because the areas studied here are ecologically vulnerable, so that the impact of acaricides may decrease the biodiversity in the zone.

In addition, capacitation programs on livestock management systems and tick control are needed. Their implementation will help farmers to make decisions that will improve livestock production. Knowing the number of animals that can be fed (system carrying capacity) from the farm’s paddocks and when animals can enter the paddocks will help to improve meat and milk production, obtaining quality products using the farm’s own resources [36,73]. Avoiding that, the deficit of pasture causes farmers to look for other grazing sites. From renting outside paddocks that increases the presence of a high level of infestation, to using entire landscape areas that are part of a forest and cause serious problems to biodiversity and soil stability [74]. 

Receiver operating characteristic (ROC) graphs are used in signal detection theory to depict the tradeoff between hit rates and false alarm rates of classifiers. This technique visualizes, organizes, and selects classifiers based on their performance [75,76]. At farm level the AUC-ROC of the OWS of high level of tick infestation was 0.72. This model, according to the Swets [76] scale is useful and can help to predict the potential level of tick infestation with an accuracy of 72.00%. Similarly, the sensitivity of the model prediction was very high for detecting farms with high levels of tick infestation. Indeed, if a farm has an OWS under 11, it has a high probability of having a low level of infestation. However, the specificity of the model was relatively low (41.00%), but this problem can be solved by a visit to the farm by a veterinarian, who can give appropriate guidance for the control of infestation, taking into account the specific context of the farm.

## 4. Materials and Methods

### 4.1. Study Area and Sampling Design

This cross-sectional survey was part of the project entitled ‘Socio-eco-epidemiology of ticks, tick-borne parasites, acaricide resistance and residual effects of acaricides in tropical Ecuadorian livestock: environmental, animal and public health impacts’. Sampling was conducted from November 2020 to March 2021 in two tropical regions of Ecuador. Area 1: Northwest of Pichincha Province in the Western Andean foothill, and Area 2: Quijos river valley in the Eastern Andean foothills.

Area 1 is located in the Northwest of Pichincha and is crossed by Chocó Andino of Pichincha Biosphere Reserve [77]. The Northwest of Pichincha is located on the western slopes of the Andes Mountains and has several altitudinal floors and microclimates [78,79,80]. Area 2 is located in the province of Napo and is located in the middle of two conservation areas, i.e., Cayambe Coca National Park and Sumaco Napo Galeras National Park [81]. Quijos river valley is located between the foothills of the eastern Andes Mountains and high jungle of the Amazon region [82].

The selection was made using snowball sampling techniques, where, with the help of community leaders and authorities, the farms were selected with an emphasis on small and medium herds. 

### 4.2. Investigation of Risk Factors Socio-Eco-Epidemiological Survey

To identify risk factors associated with high levels of tick infestation, an epidemiological questionnaire called ‘Socio-eco-epidemiological survey of ticks and TBDs’ was administered in each farm (Supplementary File S1). The survey was validated by national and international experts in the field. It was also pilot-tested on three farms in each area (one small, one medium, and one large) to ensure that farmers understood all the questions. The questionnaire was divided into four parts: (A) farm general information and herd management; (B) tick and acaricides related information; (C) inputs, outputs, and labor force used in the farm; and (D) pharmacological inputs and farming practices. This survey consisted of a personal interview with the person who knows the most about farm management, irrespective of gender or age. The data were collected using the Epicollect-5 mobile application [83], except for part D which was collected in physical form.

### 4.3. Farms Selected

The average minimum distance between farms in Quijos river valley is 1.35 km (0.08–12.80 km), and in Northwest of Pichincha 4.34 km (0.34–24.69 km). The difference in distance between each zone is due to the fact that the farms in Quijos river valley are small and medium farms, and in Northwest of Pichincha there is a majority of medium and large farms. 

According to the information collected in the survey, the farms were classified on the basis of the number of animals, as small (1 to 20 cattle), medium (21 to 70 cattle), and large (more than 70 cattle). The level of mechanization on the farm was classified using three criteria: infrastructures availability (corrals and cattle handling systems), the use of automatic or manual milking, and the usage of artificial insemination or natural services as reproduction method. A farm was considered mechanized if it met three criteria, semi-mechanized if meeting two, and non-mechanized if it only met one of the criteria.

The usage of external paddocks was considered if the paddocks used for cattle feeding were outside of the farm borders, regardless of whether the paddock was rented (paddocks of neighboring farms) or owned (paddocks of the same owner but in different locations). Paddock maintenance was defined according to two criteria: paddock topping (by scythe or machete) and paddock cleaning (feces removed). A farm performs paddock maintenance when it complies with one criterion. 

Veterinary support was evaluated in four levels according to the presence of a veterinarian on the farm: permanent, sometimes, rarely, and never. The presence of a veterinarian permanently or sometimes was classified as the presence of veterinary support. 

Knowledge on the presence of tick larvae in paddocks was a dichotomous answer ‘yes’ or ‘no’. With respect to the correct knowledge of tick location in the grass, this was classified as ‘yes’ if the farmer knows that tick larvae are located on flowers and the pasture canopy.

To identify the acaricide treatment used on the farm, acaricides were classified according to their active ingredient and the chemical group. The acaricide currently used was identified by inspection of the place where the drugs were stored and, with the help of a list of the main trade names, the acaricides used by the farmers in the 12 months prior to the visit were determined. Once the group to which the acaricide belongs was identified, the form and frequency of application, dosage, animals treated, and efficacy of the product, among others, were investigated.

### 4.4. Animals Sampled

The farms visited did not use acaricides for 10 days prior to the visit. In each farm, at least five animals were randomly selected, irrespective of breed and age. An individual health record form and general information about identification of animals (name or number), age, sex, and breed and clinical information, including a physical examination, behavior of the animal, and notations regarding observed physiological abnormalities (e.g., neoplasms and injuries), and level of tick infestation were filled out for each animal. A total of 883 individual health records were obtained.

The animals were classified according to their age into young, young adult, and adults over 7 years old. A young adult animal is between 24 and 83 months. The category adults over 7 years old, corresponds to cattle that are over the optimal culling age [84,85]. According to body condition, the animals were classified as thin (BCS 1-2), good (BCS 3), and fat (BCS 4-5), according to the body condition scale (BCS), which evaluates body condition from 1 to 5 points [86]. 

The areas where the study was carried out are dairy zones, and beef production is not significant, so this variable was not taken into account.

Color of the animals was categorized according to the most common colors present in the zones (black-white, black, brown, red, and white). Regarding breed, this was not used, because in the study areas there are no pure breeds, but rather farms use hybrids in the attempt to raise milk production. For this reason, macro groups were used: *B. primigenious taurus, Crossbreed: B. primigenious taurus* × *B. primigenious indicus,* and *B. primigenious indicus.*

### 4.5. Level of Infestation

The level of infestation of the animals was evaluated by a semi-quantitative visual inspection of the total bovine body (head, neck, back, loin, rump, arms, legs, ribs, chest, front flank, and udder) for approximately 8 min. The bovine body was divided into two parts (medial plane): right half and left half. Each half was subdivided into three zones: front third (from the head to the thoracic perimeter), the middle third (from the thoracic perimeter to the sacral bone), and the back third (from the sacral bone to the perineum). One-third was infested when it had 20 or more ingested females [87]. The presence of ticks in animals was rated in four levels: null, low, medium, and high. It was considered a low level of infestation if it was one-third infested, medium level of infestation two-thirds, and high level of infestation three-thirds infested. 

### 4.6. Morphological Identification of Ticks

The tick samples were transported to the Entomology Unit of the Institute of Research in Zoonoses, located at Central University of Ecuador in Quito. For further morphological identification, the dichotomous keys and tick species descriptions of Guerrero [88] and Barros et al. [89] were used. The specimens were identified under stereomicroscope.

### 4.7. Statistical Analyses

All data from the questionnaire were exported to Microsoft Excel^®^, to be organized and cleaned. Inconsistencies across the data base were checked and verified by the interviewer and if necessary, the farmers were contacted by phone again. For the comparison of farming and tick control practices used in the survey areas, Fisher’s exact test was used; in this case the response variable was the survey area. 

For the level of tick infestation, ordinal categorical levels (null, low, medium, and high) were assigned to a numerical scale from 0 to 3, and the average of the entire-rounded part of the five animal records with the infestation level was taken as a representation of the farm (level of tick infestation at the farm level). Farms with a score of 0 or 1 were referred to as having a low level of infestation, and with a score of 2 or 3 as having a high level of infestation. Tick infestation at the animal level was considered high when it was recorded as medium or high. Covariates with little or no variability were discarded from the analysis. Finally, a cleaned database was obtained with information of 826 animals (93.54% sampled), belonging to 139 farms (100.00% sampled).

At farm level, a univariate association test was applied, with 26 covariates. To identify the covariates for the final model, a stepwise procedure was practiced with a multiple logistic regression using the step AIC function from the MASS package [90] in the R environment [91]. For the analysis of explanatory factors at the animal level, six variables were included (age, sex, breed, color, body condition status, and lactating dairy cows). The glmer function from the lme4 package in R environment [91] was used to incorporate both fixed-effect parameters and random effects (farm to which they belong) in a linear predictor, through maximum likelihood [92]. The risk or protective explanatory variables with a *p*-Value ≤0.05 were associated with a high level of tick infestation.

A multivariate logistic model was used to combine and to evaluate the covariates. Variance inflation factor (VIF) was used to determine the degree of multicollinearity between the covariates. Function VIF from the REGCLASS package [93] in R [91] was used. If the explanatory variables were not redundant, then the VIF was equal to 1, but when the VIF value was greater than 5, they suggested the existence of multicollinearity [94,95]. 

A scoring system was developed using odds ratios (ORs) for each covariate (risk explanatory variable) in the final model. Each covariate was evaluated by its OR, and its presence/absence was coded as 1 or 0. When an OR was significantly less than one (protective explanatory variable), reverse coding of this variable was performed, its absence was recorded as 1, and the weight was 1/OR. All risk and protective explanatory variables were weighted as a single weighted overall score (OWS) by farm (farm-level risk explanatory variables) and by animal (animal-level risk explanatory variables). The area under the receiver operating characteristic curve (AUC-ROC) was used to measure performance for the OWS classification. The Youden index and ROC curve analysis were obtained by using Stata SE 14.2 [96]; likewise, this was used to estimate the best cut-off point. The Youden index was calculated to evaluate the performance of OWS in farms with high and low tick infestation levels. This Youden index was defined as sensitivity + specificity-1 [93]. The Swets [76] scale was used to qualify the usefulness of the model.

## 5. Conclusions

In conclusion, the two zones studied in the tropical part of Ecuador, had the same proportion of farms with high tick infestation, but different cattle management systems. Zone 1 of Noroccidente de Pichincha is a zone with a long history of cattle raising, better mechanization, and larger herd sizes than zone 2. Zone 2 (Quijos river valley) is a zone with a more recent establishment of livestock raising, where most farms are small and medium sized, and although it has a lower level of mechanization, they receive more support from public veterinarians. High level of infestation depends on management practices (use of amitraz with growing resistance, who prepares the acaricide solution, veterinary support, and cattle husbandry as the principal activity), infrastructure present on the farm (level of mechanization on the farm), and the usage of farm external paddocks. These factors can be considered as exploratory variables, which suggests that farmers try to generate income by practicing cattle raising as their main activity, even near natural areas. However, bad habits and practices, and lack of mechanization on the farm, can cause the level of tick infestation to be high, which makes them look for new forms of control that do not solve the problem, but cause extra expenses for production. In addition, the model found some associated factors helping to predict a high level of infestation with high sensitivity, which can contribute in a useful way to decision making on control of tick infestation.

## Figures and Tables

**Table 1:** Ticks identified in the study areas.

Tick Species	Northwest of Pichincha (Number of Farms)	Quijos River Valley (Number of Farms)	Total of Farms
*R. microplus*	63 *	67 **	130
*Ixodes boliviensis*	1 *	2 **	3
*Ixodes montoyanus*	1 *	1 **	2
*Amblyomma mixtum*	1 *	0	1

* In two farms in the Northwest of Pichincha, different species were found on the same farm (Farm 1: *R. microplus* and *A. mixtum*; Farm 2: *I. boliviensis* and *I. montoyanus);* ** In one farm in the Quijos river valley, different species were found on the same farm (*I. boliviensis* and *I. montoyanus*).

**Table 2:** Characteristics of farming and tick control in Quijos river valley and Northwest of Pichincha.

Parameter	Quijos River Valley		Northwest of Pichincha		*p*-Value of the Fisher’s Exact Test
	Number of Farms	Percentage of Farms	Number of Farms	Percentage of Farms	
Tick infestation					1.00
Low	42	58.30	40	59.70	
High	30	41.67	27	40.29	
Level of education					0.01 *
Without formal education	3	4.17	1	1.49	
Primary school	27	37.50	20	29.85	
High school ^a^	35	48.61	26	38.81	
University concluded	7	9.72	20	29.85	
Animal husbandry as principal activity	64	88.89	59	88.06	1.00
Who is the cowherd					0.07
Employee	7	9.72	6	8.96	
Owner	41	56.94	26	38.81	
Owner and Employees	24	33.33	35	52.24	
Herd size					<0.01 *
Small	28	38.89	8	11.94	
Medium	39	54.17	43	64.18	
Large	5	6.94	16	23.88	
Type of production					0.64
Beef cattle	0	0.00	1	1.49	
Dual purpose cattle	21	29.17	17	25.37	
Dairy cattle	51	70.83	49	73.13	
Level of mechanization					0.37
Non-mechanized	47	65.28	38	56.72	
Semi-mechanized	18	18.06	17	7.46	
Mechanized	7	16.67	12	35.82	
Veterinary support					<0.01 *
No	9	12.50	37	55.22	
Yes	63	87.50	30	44.78	
Acaricide					
Amide	45	62.50	58	86.57	0.08
Organophosphate	52	72.22	34	50.75	0.01 *
Pyrethroid	43	59.72	18	26.87	<0.01 *
Macrocyclic lactone	56	77.78	58	86.57	0.19
Phenylpyrazolone	1	1.39	2	2.99	0.61
Benzoylphenyl urea	8	11.11	22	32.84	<0.01 *
Pyrethroid + Organophosphate	48	66.67	32	47.76	<0.01 *
Pyrethroid + Organophosphate+ Phenylpyrazolone	22	30.56	12	17.91	0.11
Benzoylphenyl urea + Macrocyclic lactone	1	1.39	5	7.46	0.10
Benzoylphenyl urea + Phenylpyrazolone	20	27.78	16	23.88	0.70
Frequency of acaricide treatment application					0.13
Less than 1 month	44	61.11	43	64.18	
Every 1 to 2 months	21	29.17	11	16.42	
Every 3 to 6 months	6	8.33	12	17.91	
More of 6 months	1	1.39	1	1.49	

^a^ High school—including farmers with unfinished university education. * Characteristics of farming and tick control with *p*-Value ≤ 0.05.

**Table 3:** Risk and protective explanatory variables for a high level of tick infestation at the farm level using univariate analysis.

Explanatory Variable		Number of Farms	Positive Farms	Proportion	OR (95% CI)	*p*-Value of the Fisher’s Exact Test
Level of education ^a^	High School	61	26	42.62	Reference	-
	Primary School	51	19	37.25	0.80 (0.35–1.83)	0.70
	University, concluded	27	12	44.44	1.08 (0.39–2.95)	1.00
Range of experience	1–5 years	15	9	60.00	Reference	-
	6–10 years	21	8	38.10	0.42 (0.08- 1.93)	0.31
	11–20 years	25	9	36.00	0.85 (0.29–2.36)	0.82
	≥21 years	78	31	39.74	0.44 (0.12–1.56)	0.17
Who is the cowherd	Employees	13	7	53.85	Reference	-
	Owner	67	26	38.81	0.55 (0.14–2.14)	0.37
	Owner and Employees	59	24	40.68	0.59 (0.14–2.35)	0.54
Cattle husbandry as the principal activity	No	16	4	25.00	Reference	-
	Yes	123	53	43.09	2.26 (0.64–10.16)	0.19
Herd size	Large	21	8	38.10	Reference	-
	Medium	82	34	41.46	1.15 (0.39–3.57)	0.81
	Small	36	15	41.67	1.16 (0.34–4.10)	1.00
Level of mechanization	Mechanized	19	5	26.32	Reference	
	Semi-mechanized	35	15	42.86	2.07 (0.54–9.04)	0.26
	Non-mechanized	85	37	43.53	2.29 (0.71–8.82)	0.20
Cut and carry pasture	No	92	36	39.13	Reference	-
	Yes	47	21	44.68	1.25 (0.58–2.71)	0.58
Paddock maintenance	No	24	9	37.50	Reference	-
	Yes	115	48	41.74	1.19 (0.44–3.36)	0.82
Pasture rotation	No	32	15	46.88	Reference	-
	Yes	107	42	39.25	0.73 (0.31–1.76)	0.54
External paddocks	No	86	29	33.72	Reference	-
	Yes	53	28	52.83	2.19 (1.03–4.70)	0.03 *
Paddocks with dallis grass (*Paspalum dilatatum*)	No	41	17	41.46	Reference	-
	Yes	98	40	40.82	0.97 (0.44–2.20)	1.00
Knowledge of the life cycle of ticks	No	14	4	28.57	Reference	-
	Yes	125	53	42.40	1.83 (0.49–8.45)	0.4
Correct knowledge of the location of ticks in the grass	No	48	17	35.42	Reference	-
	Yes	91	40	43.96	1.43 (0.66–3.16)	0.37
Veterinary support	No	46	15	32.61	Reference	-
	Yes	93	42	45.16	1.70 (0.77–3.86)	0.2
Prescription by a veterinarian	No	37	13	35.14	Reference	-
	Yes	102	44	43.14	1.40 (0.60–3.35)	0.44
Who prepares the acaricide solution	Employed	31	16	51.61	Reference	-
	Owner	108	41	37.96	0.58 (0.24–1.39)	0.21
Person who applies acaricide treatment	Employed	48	20	41.67	Reference	-
	Owner	82	33	40.24	0.55 (0.14–2.14)	0.37
	Owner and Employees	9	4	44.44	1.12 (0.20–5.94)	1.00
Has storage area	No	83	39	46.99	Reference	-
	Yes	56	18	32.14	0.54 (0.25–1.14)	0.11
Use of amitraz	No	36	11	30.56	Reference	-
	Yes	103	46	44.66	1.83 (0.77–4.57)	0.17
Dose of acaricide	Correct	44	18	40.91	Reference	-
	Incorrect	95	39	41.05	1.00 (0.46–2.23)	1.00
Frequency of acaricide treatment application	<1 month	87	41	47.13	Reference	-
	1–2 months	32	10	31.25	0.51 (0.19–1.29)	0.15
	3–6 months	20	6	30.00	0.48 (0.14–1.50)	0.21
Perception: predisposition for a breed	No	51	15	29.41	Reference	-
	Yes	88	42	47.73	2.17 (1.00–4.93)	0.05 *
Perception: predisposition for a color	No	78	30	38.46	Reference	-
	Yes	61	27	44.26	1.27 (0.61–2.65)	0.6
Perception: predisposition for a category	No	46	18	39.13	Reference	-
	Yes	93	39	41.94	1.12 (0.52–2.48)	0.86
Perception: ticks can affect the cattle	No	11	1	9.09	Reference	-
	Yes	128	56	43.75	7.70 (1.04–342.91)	0.03 *
Perception: economic loss	No	6	1	16.67	Reference	-
	Yes	133	56	42.11	3.61 (0.39–174.87)	0.4

^a^ Primary school: including farmers without formal education; High school: including farmers with unfinished university education. ^b^ * Characteristics of farming and tick control with *p*-Value ≤ 0.05.

**Table 4:** Risk and protective explanatory variables for a high level of tick infestation at the farm level using a multivariable binary logistic regression model.

Explanatory Variables		Final Model	
		OR (95% CI)	*p*-Value of the Fisher’s Exact Test
Cattle husbandry as the principal activity	No	Reference	-
	Yes	3.96 (0.97–16.10)	0.053 ***
Level of mechanization	Mechanized	Reference	-
	Semi-mechanized	4.48 (1.02–19.53)	0.05 *
	Non-mechanized	5.11 (1.14–22.86)	0.03 *
External paddocks	No	Reference	-
	Yes	2.08 (0.94–4.60)	0.07
Veterinary support	No	Reference	-
	Yes	2.09 (0.86–5.07)	0.10
Who prepared the acaricide solution	Employee	Reference	-
	Owner	0.19 (0.06–0.61)	<0.01 **
Has storage area	No	Reference	-
	Yes	0.52 (0.23–1.20)	0.12
Use of amitraz	No	Reference	-
	Yes	2.58 (0.92–7.20)	0.07
Perception: predisposition for a breed	No	Reference	-
	Yes	1.87 (0.83–4.20)	0.13

* risk explanatory variable. ** protective explanatory variable. *** marginally significant.

**Table 5:** The probability that a farm has a low or high level of tick infestation as a function of the overall weighted score.

	Level of Tick Infestation (Number of Farms)		Total Farms	Probability of a Level of Tick Infestation	
OWS	Low	High		Low	High
5–7	4	1	5	0.80	0.20
7–9	7	0	7	1.00	0.00
9–11	13	0	13	1.00	0.00
11–13	18	11	29	0.62	0.38
13–15	24	19	43	0.56	0.44
15–17	14	23	37	0.38	0.62
17–21	2	3	5	0.40	0.60
Total	82	57	139		

Legend: for the probability, the color scale is related to the increase of its value (red to blue; with blue color being low risk and red color being high risk).

**Table 6:** Risk explanatory factors for a high level of tick infestation at the animal level using a univariate analysis.

Risk Factor		Total Animals	Positive Animals	Proportion	OR (95% CI)	*p*-Value Fisher Test
Breed	*B. p. taurus*	769	283	0.37	Reference	-
	*Crossbreed: B. p. taurus × B. p. indicus*	47	18	0.38	1.07 (0.55–2.03)	0.88
	*B. p. indicus*	10	3	0.30	0.74 (0.12–3.26)	0.75
Color ^a^	Black-White	313	122	0.39	Reference	-
	Black	142	53	0.37	0.93 (0.61–1.43)	0.76
	Brown	266	92	0.35	0.83 (0.58–1.18)	0.30
	Red	84	28	0.33	0.78 (0.45–1.33)	0.38
	White	21	9	0.43	1.17 (0.42–3.14)	0.82
Sex	Female	799	296	0.37	Reference	-
	Male	27	8	0.30	0.72 (0.27–1.74)	0.54
Age ^b^	Young	40	11	0.28	Reference	-
	Young adult	600	216	0.36	1.48 (0.70–3.36)	0.31
	Adults over 7 years old	186	77	0.41	1.86 (0.84–4.83)	0.11
Cows in lactating status	No	157	40	0.25	Reference	-
	Yes	669	264	0.39	1.91 (1.27–2.90)	<0.01 *
Body condition status	Fat	40	17	0.43	Reference	
	Good	551	191	0.35	0.72 (0.36–1.47)	0.31
	Thin	235	96	0.41	0.93 (0.45–1.97)	0.86

^a^ Color coat: classification was based according to the coat color dominance. ^b^ Age: young (cattle with ≤ 23 months); young adult (cattle between 24 to 83 months); and adults over 7 years old (cattle with ≥84 months). * risk explanatory variable.

**Table 7:** Risk and protective factors for a high level of tick infestation at the animal level included in the final multivariable binary logistic regression model.

Risk Factor		OR (95% CI)	*p*-Value Fisher test
Breed	*B. p. taurus*	Reference	-
	*Crossbreed: B. p. taurus × B. p. indicus*	0.547 (0.546–0.548)	<0.01 **
	*B. p. indicus*	0.539 (0.538–0.540)	<0.01 **
Age	Young	Reference	-
	Young adult	1.050 (1.048–1.051)	<0.01 *
	Adults over 7 years old	1.480 (1.478–1.482)	<0.01 *
Lactating dairy cows	No	Reference	-
	Yes	2.287 (2.283–2.900)	<0.01 *
Body condition status	Fat	Reference	-
	Good	1.212 (1.210–1.214)	<0.01 *
	Thin	1.992 (1.990–1.995)	<0.01 *

* risk explanatory variable. ** protective explanatory variable.

## Data Availability

The data that support the findings of this study are available from the corresponding author upon request.

## Supplementary Materials

The following supporting information can be downloaded at: https://www.mdpi.com/article/10.3390/pathogens11040403/s1, File S1: Socio-Epidemiological Survey.
