# Supplementary material for: The Associated Decision and Management Factors on Cattle Tick Level of Infestation in Two Tropical Areas of Ecuador
Source: Pathogens. 2022 Mar 26;11(4):403. doi: 10.3390/pathogens11040403 (PMC9028859; doi:10.3390/pathogens11040403)
Supplement: Supplementary file 1 [file pathogens-11-00403-s001.zip › pathogens-1616250-supplementary.pdf]

Survey Number | | | |

SOCIO-EPIDEMIOLOGICAL SURVEY: TICKS AND TBD

UNIVERSITY OF LIEGE & CATHOLIC UNIVERSITY OF LOUVAIN

CENTRAL UNIVERSITY OF ECUADOR

Zoonosis Research Institute - CIZ

The researchers request their authorization to participate in the research project entitled: Socio-eco-epidemiology of ticks, tick-borne parasites, resistance to acaricides and residual effects of acaricides in the Ecuadorian tropical cattle: impacts on the environment, public health and animal health. This project consists of: Applying a socio-eco-epidemiological survey. Collect ticks on the animal. Extract blood samples from cattle. And on certain farms collect larvae of ticks, samples of urine, feces, milk and soil. The project is committed to deliver respective results once the samples have been analyzed.

SECTION A: INTRODUCTORY SECTION

Date:_______/_____/______ **Pollster name:** ____

GPS coordinates **(gg/mm/ss): latitude**_________________**longitude**_________________ **Altitude (masl):**_____________

Farm Name:____**Name of the Owner:**____________________________________

Province Pichincha ☐ Napo ☐ **Canton** S. M. Bancos ☐ Pedro Vicente Maldonado ☐ Quito ☐ El Chaco ☐ Quijos ☐

Parish Bancos ☐ PVM ☐ Nanegal ☐ Nanegalito ☐ Gualea ☐ Pacto ☐ Baeza ☐ Borja ☐ Sardinas ☐

El Chaco ☐ **Locality:** __________________

Name of respondent: ____________________________________**Age:** **Gender:** **F** ☐ **M** ☐

Phone/Cell:_/_/_/_/_/_/_/_/_/_/

Education achieved: No formal education ☐ Primary ☐ High school ☐ Superior ☐ **Profession/Activity:**_________________

SECTION B: EXPLOITATION SYSTEM

1. **What is the total area of the farm?** ____________________________**(hectares)**

Use	Surface	Unit (m/ha)
Housing		
Cattle pen		
Pastures		
Crops		

Use	Surface	Unit (m/ha)
Forests within the property		
Temporary fallow		
Other:_______________		

2. **Are there forests outside the property? Yes** ☐ **No** ☐ How far are they from the property? ___________________

3. **Under what condition of possession does this property consist of?**
   Own ☐ Co-owner ☐ Leased ☐ Loan ☐ On departure ☐ Other:

4. **Is the owner of the farm a member of any association?**
   Yes (which)____No ☐

5. **What is the milking mode?** Manual ☐ Mechanical ☐

6. **Do you use poultry manure or other organic fertilizer as soil fertilizer?** Yes ☐ No ☐

7. **Do you use mycorrhizae to fertilize?** Yes ☐ No ☐

8. **Do you use Biol based on slurry to ferttilize?**
   Pastures ☐ Crops ☐ I don't use BIOL ☐

   *** *If you answer* *Crops, what crops?***____

9. **Is the biol used?** Prepared by you ☐ Purchased ☐

10. Are there any crops grown on your farm? Yes ☐ No ☐
*** *If you answer* **Yes,** *continue. If* **No**, *skip to question 14*

11. What is the number of varieties grown? One ☐ More than one ☐

12. Annual replacement animals

Bovines	Heads (N°)	Origin *	Price ($/animal)
Calves			
Calves			
Heifers			
First Calf Heifers			
Cows			
Herd Bulls			
Bulls			
Other			

ORIGIN: *FV= Neighboring farms; F= Fairs; 3 Other (Which)*

13. Production parameters

Parameter	Quantity	Unit
Months of lactation (average)		Months
Milk production in Dry Season		Lt/cow/day
Milk production in Rainy Season		Lt/cow/day
Average number of cows milked per month		cows/month
Average open days		Days

14. Is there a decrease in production yield in dairy cows with ticks? Yes ☐ No ☐
If your answer is Yes, in what quantity/% of the total produced:______

15. Beef cattle with ticks, how long does it take for them to reach sale weight? Age___ Sales Weight_

16. Productive Parameters (In case of beef cattle or double purpose)

Parameter	Quantity	Unit
Months of fattening (average)		Months
Average weight at deboning		Pounds
Average number of animals slaughtered annually		Animals

SECTION F: SANITARY MANAGEMENT

17. Do you move animals between these properties? Yes: ☐ No: ☐

18. Are there other lots for off-site cattle? Yes ☐ No ☐
*** *If you answer* **Yes,** *continue. If* **No**, *skip to question 32.*

19. Are these lots? Own ☐ Leased ☐

20. Are the animals moved to these lots? Constantly throughout the year ☐ In certain months ☐
what months?____

21. What category of animals are **mobilized to these lots?**____

22. What kind of grass exists in these lots?_______________________

23. After the cattle return from these lots, do they have more or less ticks? More ☐ Less ☐

24. How long the animals spend in these lots?_____________________

25. Do you perform veterinary support? Permanent ☐ Sometimes ☐ Never ☐ Hardly ever ☐

26. The veterinarian that provides technique attendance in your farm is? Private ☐ GAD ☐ MAG ☐

27. Who prescribes the treatment?
Owner ☐ Veterinarian ☐ Farm Store Salesman ☐

Butler ☐ Workers ☐ Visitor ☐ Other______

28. Who applies the treatments?

Owner ☐ Veterinarian ☐ Butler ☐ Workers ☐ Other______

29. Which of these diseases have you observed in your livestock?

Illness	N° animals affected/year	Mortality		Categories of cattle concerned
		No	%	
Internal parasitosis				
Tick fever				
Abortion				
Mastitis				
Metritis				
Placental retention				
Milk fever				
Dystocic labor				
Pneumonia				
Tympanism				
Myiasis				
Nuche (tupe)				
Abscess				
Lameness				
Heat stroke				

30. What do you think is the degree of affection that ticks cause in your animals?

None ☐ Low ☐ Middle ☐ High ☐

31. Did you know that ticks can transmit disease? Yes ☐ No ☐

What diseases?____

32. Which breeds of animals on your farm have been the most affected by ticks? Breeds:________________

33. Do you identify any of them?

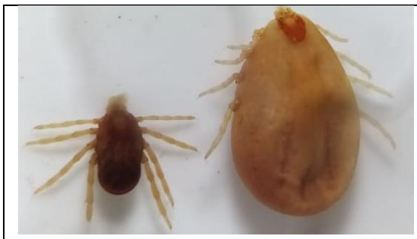

Rhipicephalus microplus

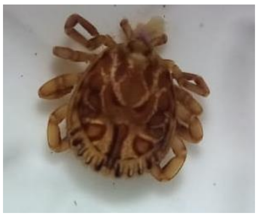

Amblyomma cajennense

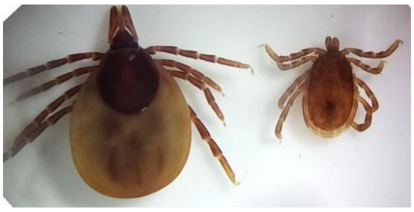

Ixodes boliviensis

34. In which third of the bovine body are ticks most frequent?

Previous Third ☐ Middle Third ☐ Back Third ☐ All ☐

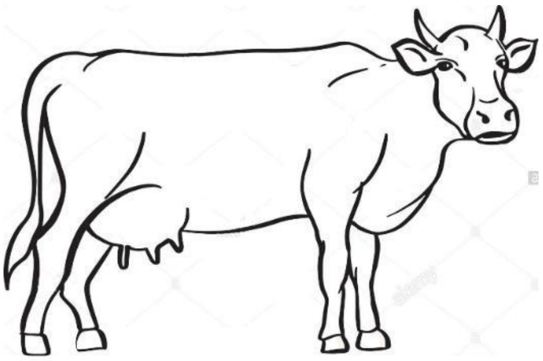

35. **When are there more ticks on the cattle on your farm?**
Rainy season ☐ All year round ☐ Dry season ☐
Specify in which months:____

36. **Do you use chemical remedies to control ectoparasites in livestock on your farm?** Yes ☐ No ☐
***** *If you answer **Yes,** continue. If **No**, skip to question 51*

37. **Do you calibrate the spraying equipment? Yes** ☐ **No** ☐
Every so often?______________________

38. **Who prepares the solutions with the acaricide?** Veterinarian ☐ Owner ☐ Workers ☐

39. **What water do you use to dissolve the acaricide?**
Drinking water ☐ Irrigation ☐ River ☐ Bottled water ☐

40. **What are the rotation dynamics of the acaricides used?**____
____

41. **Which of the following acaricides do you use or have you used on your farm?**

Organophosphates:

**Trade name**	**Frequency of use**	**Application**	**This product was......**
Nuvan	Less than one month	In immersion baths	Lately used
Neguvon	Between 1 and 2 months	By spraying with pump	Used some time ago
Asuntol	Between 3 and 6 months	Injected	Uses for a long time
Diclorvos	More than 6 months	Pour on	**Treated animals**
Garafos	**Dosage:**	Cleaning with cleaner	All Only those affected
Matanuche	**Duration of Tto.**	**Retirement time**	Dry cattle Calves
	**Efficacy (%)**	Milk Meat	Bulls
**What do I use before this product?**		**How long did you use it?**	

Pyrethroids

**Trade name**	**Frequency of use**	**Application**	**This product was......**
Nuvan	Less than one month	In immersion baths	Lately used
Neguvon	Between 1 and 2 months	By spraying with pump	Used some time ago
Asuntol	Between 3 and 6 months	Injected	Uses for a long time
Diclorvos	More than 6 months	Pour on	**Treated animals**
Garafos	**Dosage:**	Cleaning with cleaner	All Only those affected
Matanuche	**Duration of Tto.**	**Retirement time**	**Retirement time** Calves
	**Efficacy (%)**	Milk Milk	Bulls
**What do I use before this product?**		**How long did you use it?**	

Amitraz

**Trade name**	**Frequency of use**	**Application**	**This product was......**	
Nuvan	Less than one month	In immersion baths	Lately used	
Neguvon	Between 1 and 2 months	By spraying with pump	Used some time ago	
Asuntol	Between 3 and 6 months	Injected	Uses for a long time	
Diclorvos	More than 6 months	Pour on	**Treated animals**	
Garafos	**Dosage:**	Cleaning with cleaner	All	Only those affected
Matanuche	**Duration of Tto.**	**Retirement time**	**Retirement time**	Calves
	**Efficacy (%)**	Milk Milk	Bulls	
**What do I use before this product?**		**How long did you use it?**		

Ivermectins

**Trade name**	**Frequency of use**	**Application**	**This product was......**	
Nuvan	Less than one month	In immersion baths	Lately used	
Neguvon	Between 1 and 2 months	By spraying with pump	Used some time ago	
Asuntol	Between 3 and 6 months	Injected	Uses for a long time	
Diclorvos	More than 6 months	Pour on	**Treated animals**	
Garafos	**Dosage:**	Cleaning with cleaner	All	Only those affected
Matanuche	**Duration of Tto.**	**Retirement time**	**Retirement time**	Calves
	**Efficacy (%)**	Milk Milk	Bulls	
**What do I use before this product?**		**How long did you use it?**		

Fipronil

**Trade name**	**Frequency of use**	**Application**	**This product was......**	
Nuvan	Less than one month	In immersion baths	Lately used	
Neguvon	Between 1 and 2 months	By spraying with pump	Used some time ago	
Asuntol	Between 3 and 6 months	Injected	Uses for a long time	
Diclorvos	More than 6 months	Pour on	**Treated animals**	
Garafos	**Dosage:**	Cleaning with cleaner	All	Only those affected
Matanuche	**Duration of Tto.**	**Retirement time**	**Retirement time**	Calves
	**Efficacy (%)**	Milk Milk	Bulls	
**What do I use before this product?**		**How long did you use it?**		

Luazuron (Chitin Inhibitor)

**Trade name**	**Frequency of use**	**Application**	**This product was......**	
Nuvan	Less than one month	In immersion baths	Lately used	
Neguvon	Between 1 and 2 months	By spraying with pump	Used some time ago	
Asuntol	Between 3 and 6 months	Injected	Uses for a long time	
Diclorvos	More than 6 months	Pour on	**Treated animals**	
Garafos	**Dosage:**	Cleaning with cleaner	All	Only those affected
Matanuche	**Duration of Tto.**	**Retirement time**	**Retirement time**	Calves
	**Efficacy (%)**	Milk Milk	Bulls	
**What do I use before this product?**		**How long did you use it?**		

Combinados Amitraz-Piretroides

**Trade name**	**Frequency of use**	**Application**	**This product was......**
Nuvan	Less than one month	In immersion baths	Lately used
Neguvon	Between 1 and 2 months	By spraying with pump	Used some time ago

Asuntol	Between 3 and 6 months	Injected	Uses for a long time
Diclorvos	More than 6 months	Pour on	**Treated animals**
Garafos	**Dosage:**	Cleaning with cleaner	All Only those affected
Matanuche	**Duration of Tto.**	**Retirement time**	**Retirement time** Calves
	**Efficacy (%)**	Milk Milk	Bulls
**What do I use before this product?**		**How long did you use it?**	

Combinados Piretroides – O**rganophosphates**

**Trade name**	**Frequency of use**	**Application**	**This product was......**
Nuvan	Less than one month	In immersion baths	Lately used
Neguvon	Between 1 and 2 months	By spraying with pump	Used some time ago
Asuntol	Between 3 and 6 months	Injected	Uses for a long time
Diclorvos	More than 6 months	Pour on	**Treated animals**
Garafos	**Dosage:**	Cleaning with cleaner	All Only those affected
Matanuche	**Duration of Tto.**	**Retirement time**	**Retirement time** Calves
	**Efficacy (%)**	Milk Milk	Bulls
**What do I use before this product?**		**How long did you use it?**	

Combinados Piretroides – O**rganophosphates-Fipronil**

**Trade name**	**Frequency of use**	**Application**	**This product was......**
Nuvan	Less than one month	In immersion baths	Lately used
Neguvon	Between 1 and 2 months	By spraying with pump	Used some time ago
Asuntol	Between 3 and 6 months	Injected	Uses for a long time
Diclorvos	More than 6 months	Pour on	**Treated animals**
Garafos	**Dosage:**	Cleaning with cleaner	All Only those affected
Matanuche	**Duration of Tto.**	**Retirement time**	**Retirement time** Calves
	**Efficacy (%)**	Milk Milk	Bulls
**What do I use before this product?**		**How long did you use it?**	

42. **Where do you purchase used acaricides?** Visitor ☐ Informal market ☐ Agricultural store ☐
43. **Products used have sanitary registration (Verification)** Yes ☐ No ☐
44. **Do you use any alternative methods for tick control?**

Use of birds, insects and/or fungi	☐	Flooding of pastures	☐
Pasture rotation	☐	Burning of pastures	☐
Baths with medicinal plants	☐	Transhumance	☐
Planting repellent plants	☐	Spraying of Garrapaticides on pastures	☐
Use of burned oil (Kerosene)	☐	Vaccination	☐
Crossbreeding of breeds	☐	Sulphur baths	☐

Products used: Brief description and frequency of the method used:____

SECTION C: PRODUCTION

45. What is the cattle production aptitude of your farm?

Milk ☐ Meat ☐ Mixed or Double Purpose ☐

46.

47. Destination of milk production

Destiny	Quantity (lt)	Frequency of sale	Price ($/lt)
Regional dairy plant			
Local dairy plant			
Cheese factories			
Farm industrialization			
Calf rearing			NOT APPLICABLE
Human consumption			NOT APPLICABLE
Direct sale			
Other destination:			

In case of industrialization on the property:

Product	Quantity of milk used (lt)	N° Product obtained	Frequency of production	Sale price ($/Unit)

48. Sale of Beef Cattle

	N° Heads Sold	Frequency of sale	Average weight at the post or sale (kg)	Destination	Price ($/Unit)
Calf for fattening					
Steer for fattening					
Cows/ Replacement Heifers					
Cows-Culled					
Finished Cattle					
Bulls-Culled					

UNIT: *Animal-Pound-Kilogram*

49. In case you have pigs/poultry/fish answer:

Category	Sale standing			To retailer on premises (foot)			Direct sales in pounds			Butcher´s		
	No	Frequency	$/Unit	No	Frequency	$/Unit	No	Frequency	$/Unit	No	Frequency	$/Unit
Piglets												
Sows												
Fattening pigs												
Boars												

Category	Sale standing			To retailer on premises (foot)			Direct sales in pounds			Butcher´s		
	No	Frequency	$/Unit	No	Frequency	$/Unit	No	Frequency	$/Unit	No	Frequency	$/Unit
Layers												
Breeders												
Fattening												

Number of fish harvested	Selfconsumption (lb)	Sale

(monthly)	No	lb	($/lb)

SECTION D: LABOR, CONSUMABLES AND MISCELLANEOUS EXPENSES

50. Is there family labor in day-to-day tasks?**?** Yes ☐ No
***** *If you answer **Yes,** continue. If **No**, skip to question 58*

51. Family composition and occupation

**Family** Relationship	**Age (years)**	**Sex (F/M)**	**Main activity on the farm**	**When?**		**Do you get paid for the work done?**		**Activities outside the farm**	**Payment/Income ($/Frequency*)**
				Hours to day	Days to the Week	No	Yes (Payment $/Frequency)		

*Frequency: Monthly-Weekly-Daily

52. Are the workers on your farm? Occasional ☐ Permanent ☐
***** *If you answer **Permanent,** continue. If you answer **Occasional,** skip to question 60*

53. Compensation of workers

**N° of workers**	**Hours worked per day**	**Days worked per month**	**Pay ($/Frequency)**	**Extra bonus**

*Frequency: Monthly-Weekly-Daily

54. Who performs these internships?

**Activity**	**N° of animals**	**Frequency**	**Persons performing the activity**			**Time spent (Hours/Frequency)**	**Payment ($/Unit***)**
			**Sexo (F/M)**	**Who?****	**N° of people by activity**		
**LIVESTOCK MANAGEMENT**							
Cattle branding/marking							
Dehorning							
Castration							
Weight registration							
Herding cattle							
Milking cows							
**SANITARY CONTROL**							
Bathing Cattle (Ticks)							
Manual removal of ticks							
Wipe the animal with a cleaner with tick killer.							
Deworming							
Vaccination							
Diagnosis/treatment of disease (Veterinary care)							

**REPRODUCTIVE ACTIVITIES**							
Insemination							
Synchronize heats							
Pregnancy test (palpation)							
Pregnancy test (ultrasound)							
Other______________________							
**INSTALLATION MAINTENANCE**							
Raising fences	NOT APPLICABLE						
Cleaning of corrals	NOT APPLICABLE						
Cleaning of paddocks	NOT APPLICABLE						
Cleaning of milking parlor	NOT APPLICABLE						
**FEEDING**							
Silage	NOT APPLICABLE						
Haymaking	NOT APPLICABLE						
cut and carry pasture	NOT APPLICABLE						
Offer feed/by-products							
Feeding calves							
Other:							
*Frequency: Monthly-Weekly-Daily **Who: Veterinarian-Butler-Worker-Owner ***Unit: Daily-Per Visit- Per N° Animals							

55. Procurement of inputs.

Input	Name	Quantity	Frequency of purchase*	Cost ($/unit)
Detergents				
Sealants				
Mastitis Test				
Drying towels				
Semen				
Analgesic				
Antibiotics				
Disinfectants				
Healing				
Hormones				
Minerals (Injectable)				
Vaccines				
Vitamins				
Internal and external				

dewormers				
Restoratives (serums)				
Fertilizers				
Phytosanitary products				

*Frequency: Annual-Monthly-Weekly-Daily

56. Food supplement

Supplement	Quantity Purchased	Frequency purchase	Cost ($/Unit)
Balanced cows			
Calf feed			
Overfeed			
Calf substitute			
Mineral salts			
Banana rejection			
Palm heart rejection			
Silo			
Hay			
Other................			

57. Annual farm costs

Item	Quantity	Estimated Value ($/Unit)	Years of useful life	Years in use	Maintenance cost ($/Frequency)
Milking equipment					
Cooling tank					
Corrals/Stablos/Mangas					
Cellar					
Electric fence					
Tractor					
Irrigation equipment					
Fumigation pump					
Scythe					
Grinder					
Biodigester					
Water pump					
Others:					

58. Payments to third parties

Item	Cost	Frequency
Electricity		
Water		
Mobilization		
Laboratory services		
Land/pasture lease payment		

Payment of agricultural loans		

*Frequency: Annual-Monthly-Quarterly

59. Has your property suffered from cattle rustling this year? Yes ☐ No ☐
Heads per year___________________________________

60. Do you think the presence of ticks causes economic losses in your herd? Yes ☐ No ☐

61. What were the changes in your livestock and/or agricultural system due to the Covid-19 pandemic? _______________
____

62. Based on the practices this exploitation is: Extensive ☐ Intensive ☐ Semi intensive ☐
